# Application Progress of Polyaniline, Polypyrrole and Polythiophene in Lithium-Sulfur Batteries

**DOI:** 10.3390/polym12020331

**Published:** 2020-02-05

**Authors:** Xiaodong Hong, Yue Liu, Yang Li, Xu Wang, Jiawei Fu, Xuelei Wang

**Affiliations:** 1 School of Materials Science and Energy Engineering, Foshan University, Foshan 528000, China; 2 College of Materials Science and Engineering, Liaoning Technical University, Fuxin 123000, China; liuyue471804@163.com (Y.L.); lb622975@163.com (Y.L.); hxd9917@126.com (X.W.);

**Keywords:** Li-S batteries, sulfur confinement, polyaniline, polypyrrole, polythiophene

## Abstract

With the urgent requirement for high-performance rechargeable Li-S batteries, besides various carbon materials and metal compounds, lots of conducting polymers have been developed and used as components in Li-S batteries. In this review, the synthesis of polyaniline (PANI), polypyrrole (PPy) and polythiophene (PTh) is introduced briefly. Then, the application progress of the three conducting polymers is summarized according to the function in Li-S batteries, including coating layers, conductive hosts, sulfur-containing compounds, separator modifier/functional interlayer, binder and current collector. Finally, according to the current problems of conducting polymers, some practical strategies and potential research directions are put forward. We expect that this review will provide novel design ideas to develop conducting polymer-containing high-performance Li-S batteries.

## 1. Introduction

The rapid development of industry and the massive consumption of fossil energies aggravate global climate change and environmental pollution, which has produced a common research theme about developing renewable energies involving solar energy, wind energy, tide energy, and chemical energy, including supercapacitors and batteries. With the popularization of electric vehicles, traditional lithium-ion batteries cannot satisfy the urgent requirement of high endurance mileage. Lithium-sulfur (Li-S) batteries are widely considered as the next-generation high-performance energy storage devices beyond lithium-ion batteries [1], due to their high theoretical capacity (1675 mAh g^−1^) and energy density (~2500 Wh kg^−1^). However, the industrialization of Li-S batteries has been hindered by several reasons: (i) The insulation features of sulfur restrict its wide application, and conductive agents are needed to improve the conductivity of sulfur-containing cathodes; (ii) The volume change (~80%) caused by the transformation between solid S_8_ and dissolved lithium polysulfides (LiPSs) destroys the internal structure of electrode materials; (iii) The shuttle effect of dissolved LiPSs deteriorates the long-term cycling performance of Li-S batteries. In order to overcome the insulation features of sulfur, carbon-based materials including porous carbon, carbon nanotubes, carbon fibers and graphene or reduced graphene oxide (rGO), have been adopted as conductive agents or substrates to host sulfur [2]. To a certain extent, the problems of poor conductivity and volume change can be overcome by increasing the conductivity of sulfur-containing cathodes and the specific surface area or pore distribution. Besides physical methods, chemical anchoring and electrocatalytic strategies have been widely reported in designing sulfur host materials and separator modifiers in recent years [3]. For example, various metal oxides, hydroxides, sulfides, carbides, nitrides and polymers have been adopted to improve the long-term cycling performance of Li-S batteries [4]. On the other hand, the electrocatalytic strategy in Li-S batteries aims to improve the sluggish reaction kinetics between solid S_8_, Li_2_S and dissolved long-chain LiPSs [5]. Through catalyzing the reversible conversion of sulfur species or accelerating the transfer rate of Li^+^ ions, the electrochemical performance of Li-S batteries can be comprehensively improved.

Recently, carbon-based materials and metal compounds are the main research hotspots in Li-S batteries. Compared to the widely reported carbon-based materials and metal compounds, conducting polymers have received little attention in the field of Li-S batteries [6]. However, traditional conducting polymers including polyaniline (PANI), polypyrrole (PPy), poly(3,4-ethylenedioxythiophene) (PEDOT), and PEDOT:poly(4-styrene sulfonate) (PEDOT:PSS), are famous for the facile preparation, high flexibility, and high electrical conductivity (up to 500 S cm^−1^) [7]. In addition, N-containing functional groups and *p*-conjugated structures in the backbone of conducting polymers can be adopted as active sites or reactive groups to confine the sulfur species. Therefore, these conducting polymers are often used as coating layers, conductive hosts, separator modifier/functional interlayers and binders for Li-S batteries. For example, after wrapping with conducting polymers layers, the conductivity of sulfur cathodes is improved, meanwhile, the diffusion and shuttling of lithium polysulfides are also suppressed effectively by the N-containing groups in polymers. Especially for the commercialization of conducting PEDOT: PSS solutions, it is very convenient to prepare conducting coatings on the surface of powders. Therefore, most works in Li-S batteries have been focused on the conducting coating layers or core-shell structures. In addition, the binders of conducting polymers also integrate the conducting feature with the sticky behavior of polymers, which exhibits a superior performance than that of traditional PTFE or PVDF binders. When serving as the separator modifier or functional interlayer, the conductive layers are often placed on the separator surface or between the cathode and separator to retard the shuttling of dissolved polysulfides by physical obstruction and/or chemical adsorption.

In view of these advantages of conducting polymers [8], PANI, PPy and PTh have been utilized as the components for Li-S batteries. In this review, we classify the function of conducting polymers into coating layer, conductive host, separator modifier/interlayer, current collector and binder, and the application progress of each conducting polymer is summarized in detail. Finally, according to the current problems of conducting polymers in Li-S batteries, we put forward some practical strategies, and expect that the review will provide novel design ideas for researchers in the field.

## 2. Application of PANI in Li-S Batteries

PANI is a popular conducting polymer, which can be easily synthesized by chemical oxidative methods [9], interfacial polymerization [10], electrochemical methods [11,12], template methods [13], hydrothermal methods [14], and so on. By using these methods, PANI nanofibers [15], hollow spheres [16], and three-dimensional (3D) cross-linked PANI [17] can be synthesized successfully, and utilized as different components in Li-S batteries, including coating layer, conductive host, covalent bond compounds, separator modifier/interlayer, and redox mediator.

### 2.1. Coating Layer

**Figure 1:**
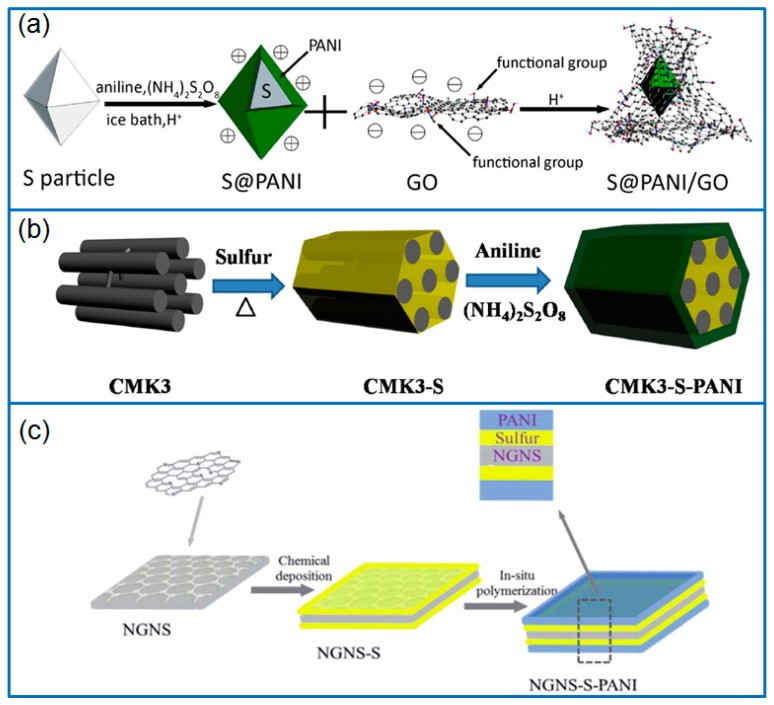
(**a**) The preparation process of S@PANI/GO [19]; (**b**) The wrapping process of CMK3/S-PANI composite [23]; (**c**) Preparation of NGNS-S-PANI [24].

In view of the electronic and ionic conducting feature, PANI wrapping layers are often generated on the surface of sulfur or sulfur/carbon composites to improve the long-term cycling performance of Li-S batteries by overcoming the insulation properties of sulfur. In the field of sulfur particle coating, Duan et al. [18] firstly wrapped sulfur particles with poly(allylamine hydrochloride) (PAH) and opposite charged poly(styrenesulfonate sodium salt) (PSS) alternately by a layer-by-layer assembly, then generated a PANI layer on polymer-coated sulfur (PAH/PSS@S) by in-situ polymerization. In another work, PANI and GO both acted as coating layers to wrap bipyramidal sulfur particles [19], as shown in Figure 1a. The inner PANI layer improved the conductivity of sulfur particles, and the GO outer layer served as a buffer to overcome the volume change. Moreover, PANI/GO double shells also confined the dissolution and diffusion of dissolved lithium polysulfides (LiPSs) by physical and chemical interaction. The S@PANI/GO cathode retained a capacity of 641 mAh g^−1^ at 1 C after cycling for 300 cycles, which was much better than that of the single layer wrapped sulfur cathode (S/GO or S@PANI). An et al. [20] prepared a dual-shell hollow PANI/S-core/PANI composite (hPANI/S/PANI) by using silicon spheres as template. In this nanostructure, S particles were deposited on the surface of PANI and distributed between two PANI layers. Double PANI layers facilitated the ion permeation, overcame the volume expansion and inhibited the diffusion of the polysulfides. The hPANI/S/PANI cathode exhibited an improved cycling performance, and the capacity was maintained at 572.2 mAh g^−1^ after 214 cycles at 0.1 C.

Aside from coating sulfur particles directly, PANI has been used for wrapping porous carbon/S composites. For example, acetylene black was used as porous carbon to deposit sulfur, and then aniline monomers were polymerized on the surface of the S-C composite to prepare core/shell structured PANI@S-C composites [21]. The PANI shell effectively enhanced the conductivity of electrode materials, alleviated the volume expansion and trapped LiPSs by electrostatic interaction. An optimized PANI@S-C composite cathode with 12.5 wt.% PANI exhibited the optimum electrochemical performance, with an initial discharge capacity of 1257 mAh g^−1^ at 0.16 mA cm^−2^. In another work, conductive PANI was grafted on acetylene black particles, and sulfur particles were chemically deposited on the C-PANI particles to form C-PANI-S particle congeries. In the last step, PANI was polymerized on C-PANI-S particle congeries to prepare a multi-core-shell structure [22]. The C-PANI-S@PANI composite achieved a high sulfur content of 87 wt.%, and the areal density of sulfur was above 6 mg cm^−2^. The wrapped composite delivered an initial capacity of 1011 mAh g^−1^, and the capacity remained at 835 mA h g^−1^ after cycling for 100 cycles at 0.2 C.

Regarding the design of porous carbon matrices, Jin et al. [23] prepared PANI-wrapped mesoporous carbon/sulfur (CMK3/S-PANI) composites by the in-situ polymerization method shown in Figure 1b. As a micro-reactor, mesoporous carbon CMK3 ensured the excellent conductivity and provided enough space for the volume change of sulfur species, while the PANI layer physically retarded the dissolution of LiPSs. Therefore, the porous structure and PANI coating provided double barriers to alleviate the shuttling of dissolved LiPSs. As a result, the PANI coated CMK3/S cathode exhibited an initial capacity of 1103 mAh g^−1^, and remained at 649 mAh g^−1^ at 1 C after 100 cycles. Ding et al. [25] prepared a spherical ordered mesoporous carbon/sulfur composite (S-OMC/S) firstly, and then wrapped a PANI layer on the surface of S-OMC/S particles. The mesoporous structure and conductive PANI coating effectively improved the capacity and cycling performance of Li-S batteries. The PANI@S-OMC/S cathode exhibited a high initial capacity of 1626 mAh g^−1^ at 0.1 C, and the capacity still remained at 1338 mAh g^−1^ after cycling for 100 cycles. In addition, Hu et al. [26] prepared a PANI coated sulfur-embedded colloid carbon spheres cathode with a sulfur content of 73 wt.%. The colloidal carbon sphere and PANI outer layer improved the electron transfer rate, buffered the volume change of S, and prevented the diffusion of dissolved LiPSs. Therefore, it achieved a super-long cycling performance for 2500 cycles at 5 C, with a low capacity decay of 0.01% cycle^−1^. In addition, Wu et al. [27] synthesized yolk-shell hierarchical porous carbon (HPC) nanospheres with mesoporous core and microporous shell as sulfur hosts. In order to improve the sulfur confinement efficiency, the sulfur infused yolk-shell HPC (HPC@S) was glued by a PANI coating in the presence of carbon nanotubes (CNTs). Compared to conventional “point-to-point” transport mode, the CNT-decorated PANI layer achieved a “point-to-plane’’ type electron transport by forming a conductive network. Due to the confinement of CNT-decorated PANI coating, the shuttling of dissolved LiPSs was efficiently inhibited, and the cathode exhibited a high initial capacity of 1372 mAh g^−1^ at 0.2 A g^−1^. The capacity decay was low as 0.083% cycle^−1^ for 500 cycles at 2 A g^−1^.

In addition to various porous carbon materials, one-dimensional carbon nanotubes and carbon nanofibers have an excellent electronic conductivity, and can be adopted as sulfur hosts in Li-S batteries. The encapsulation of conductive PANI layer on carbon nanotube/S or carbon nanofiber/S will efficiently suppress the diffusion of dissolved polysulfides and further enhance the cycling stability of Li-S batteries. For example, Li et al. [28] deposited sulfur on multi-walled carbon nanotubes (MWCNTs-S), then wrapped a PANI layer on the surface of MWCNT-S by using ascorbic acid as dopant and inhibitor to form sandwich-like MWCNTs-S@PANI composites. The MWCNTs-S@PANI cathode delivered a higher discharge capacity, better cyclic performance and rate capability than that of MWCNTs-S cathode. Kim et al. [29] prepared a sulfur-infused single-walled carbon nanotube (S/SWNT) film firstly, then immersed the free-standing S/SWNT film into an aniline solution to prepare PANI-coated S/SWNT films. As an integrated cathode without current collector, the PANI-S/SWNT cathode delivered a capacity of 1011 mAh g^−1^ at 0.2 C at the 100th cycle. The capacity retention and rate performance were much higher than that of a S/SWNT cathode, which was attributed to the sulfur immobilization role of the PANI coating.

Similar to SWNT films, a three-dimensional (3D) conductive carbon nanofibers (CNFs) mat can serve as substrate. After melting with sulfur, PANI was polymerized on a CNF/S substrate to fabricate a free-standing CNF/S/PANI cathode [30]. The interconnected CNF/PANI network promoted the fast transfer of electrons, and buffered the volume expansion of sulfur. Moreover, the oxygen and nitrogen heteroatoms in the network also provided abundant anchoring sites to adsorb polysulfides. As a result, a PANI-wrapped 3D CNF/S cathode exhibited a long-term cycling stability, and the capacity decay was low as 0.08% cycle^−1^ for over 300 cycles.

Among carbon materials, GO has been widely used to host sulfur in Li-S batteries. However, the PANI wrapping on GO/S composite also improves the cell performance. In this field, based on layer-by-layer assembly and heat treatment, Moon et al. [31] prepared a cross-linked PANI layer on a GO-S composite to produce a GO-S@PANI cathode. Under the protection of the crosslinked PANI layer, the dissolution of polysulfides was effectively inhibited. Even at a high sulfur content of 75 wt.%, the capacity retention of Li-S cell remained at 80.43% after 500 cycles at 1 C. Qiu et al. [32] prepared a cetyltrimethylammonium bromide (CTAB)-GO-S composite by a chemical deposition method, then polymerized PANI in-situ on CTAB-GO-S composite. The nitrogen-containing groups of the PANI layer effectively trapped the dissolved LiPSs, which improved the long-term cycling performance of the Li-S cell. The PANI-modified CTAB-GO-S cathode achieved a stable cycling for over 500 cycles, with a capacity decay of 0.051% cycle^−1^ at 1 C. In addition, Ding et al. [24] designed an uniform PANI coating on a nitrogen-doped graphene/sulfur (NGNS-S) layered structure by two-step method as illustrated in Figure 1c. The PANI elastic layer effectively avoided the shuttling of LiPSs and buffered the volume expansion of sulfur. Meanwhile, nitrogen-doped graphene ensured the electrical conductivity of the electrode. This ternary layered NGNS-S-PANI cathode delivered a better electrochemical performance than that of NGNS-S cathode, with a retained capacity of 693 mA h g^−1^ for 100 cycles at 0.5 C.

### 2.2. Conductive Host

PANI not only acts as a wrapping layer for sulfur and carbon/sulfur composites, but also serves as an excellent conductive matrix to host sulfur. For instance, Lu et al. [33] synthesized a 3D urchin-like S/PANI composite with a sulfur content of 55 wt.%, in which, aniline was polymerized in-situ on as-produced sulfur particles. The conductive PANI layer on sulfur endowed a superior conductivity to the composite. When assembled in a Li-S cell without any conducting agent, the novel S/PANI cathode delivered a high initial capacity (1095 mAh g^−1^ at 0.1 C) and a good rate capability. In another work, the surface of PANI nanofibers was deposited with thin 10 nm sulfur layer to form a S-PANI composite [34]. The positively charged PANI nanofiber not only transferred electrons like carbon materials, but only efficiently adsorbed negatively-charged polysulfides. As a result, the S-PANI cathode delivered a high initial capacity of 977 mAh g^−1^, and the capacity remained at 88.3% after 100 cycles (1 C). In addition, PANI can be used to wrap mesoporous carbon Ketjen black (KB), and then host sulfur to prepare PANI-coated KB carbon/S composite cathodes [35]. The optimized PANI coating (30 wt.%) enhanced the contact between sulfur and the carbon matrix by providing a conductive link. Moreover, the PANI layer also improved the cycling stability of Li-S cell. In another work, PANI was used to coat commercial carbon black (PANI-C) as a sulfur host [36]. As-prepared PANI-C composite containing 20 wt.% PANI exhibited a large specific surface area and a high conductivity of 30 S cm^−1^. Then, sulfur was melted into the porous PANI-C host by a two-step heat treatment. The rate capability and cycle stability of the Li-S battery was improved greatly, which was attributed to the high conductivity of PANI-C host. Moreover, the flexible PANI improved the contact between carbon and sulfur, and overcome the loss of active sulfur species. In another work, PANI was grown on oxidized carbon black to fabricate a conducting polymer spherical network (PSN) [37]. Then, sulfur was infused into the pores of the PSN framework to produce a C-S@PANI composite. The PANI conducting network facilitated the conduction of electrons and lithium ions, and the S-C covalent bonds produced by heat treatment effectively immobilized sulfur species. Due to the special structure of PSN framework, the C-S@PANI cathode exhibited a superior cycling stability at 50 and 0 °C, and the capacity remained at 922 and 581 mAh g^−1^, respectively, after 200 cycles.

**Figure 2:**
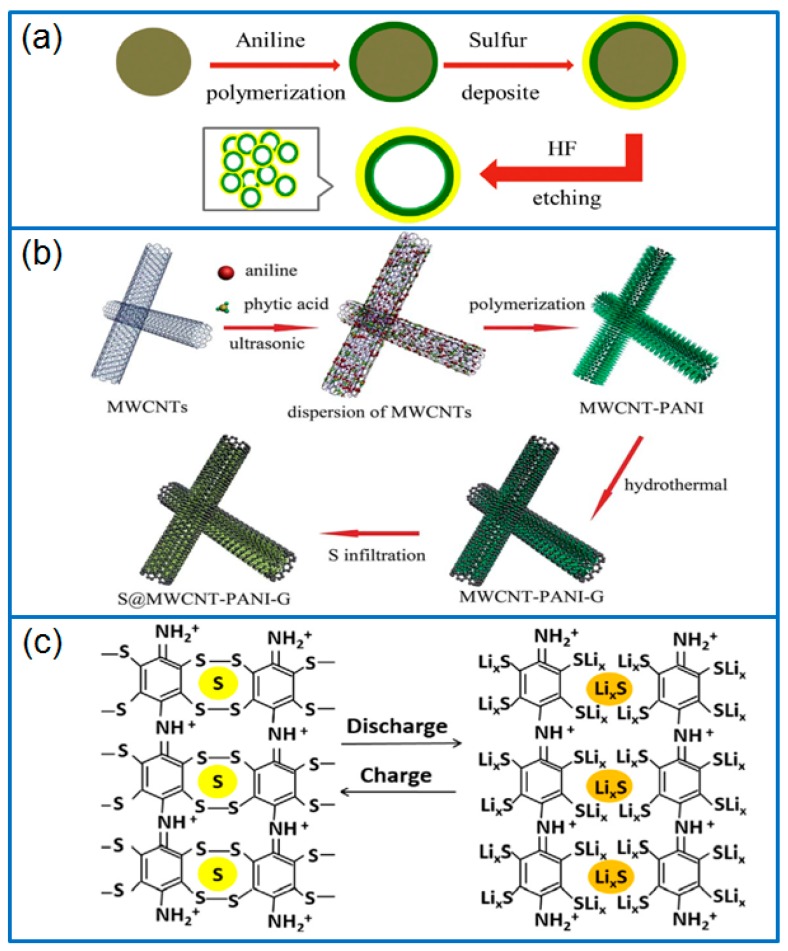
Schematic presentation of preparing (**a**) hPANIs@S composite [39], (**b**) S@MWCNT-PANI-G composite [40], and (**c**) SPANI compound cathode [44].

Ma et al. [38] fabricated PANI hollow spheres by a vapor phase infusion method, and then melted sulfur on the inner and outer surface of hollow spheres to prepare a hollow PANI-S composite cathode. The void space of the hollow spheres buffered the volume change of sulfur. Furthermore, the generated S-C bonds between sulfur and PANI during heating treatment effectively inhibited the shuttle effect of Li-S batteries. The spherical host of conductive PANI further ensured the long-term cycling performance of Li-S batteries. Wei et al. [39] synthesized PANI on silicon spheres, and chemically deposited sulfur on PANI. After etching the silicon spheres, a hollow PANI spheres (hPANIs)@S composite (Figure 2a) was obtained with sulfur dispersed on hPANIs uniformly. As a cathode in Li-S battery, the capacity was maintained at 601.9 mAh g^−1^ after 100 cycles at 170 mA g^−1^. Deng et al. [40] polymerized a PANI layer on multi-wall carbon nanotubes (MWCNTs) to absorb dissolved LiPSs through physical and chemical interaction, then wrapped a graphene (G) layer by a hydrothermal method. Three-component MWCNT-PANI-G composite (Figure 2b) was used as an excellent sulfur host to improve the electrochemical performance of Li-S batteries. Even at a high sulfur content of 68 wt.%, the Li-S cell exhibited a high initial capacity and a good rate capability. In another work, Zhang et al. [41] polymerized PANI in-situ on hollow carbon nanofiber (HCNF) to prepare a core-shell structured HCNF@PANI composite, and then infiltrated sulfur into the HCNF@PANI composite by heat treatment. As a conductive host, the PANI layer accommodated active sulfur, even reaching a sulfur content of 74.4 wt.%. Due to the good dispersion of sulfur in HCNF@PANI, the HCNF@PANI-S cathode exhibited an initial capacity of 960 mAh g^−1^, and remained at 535 mAh g^−1^ after 200 cycles (0.5 C).

Besides carbon nanotubes/carbon nanofibers, PANI can also be deposited on graphene sheets to serve as composite sulfur hosts. For example, PANI was polymerized on graphene nanoribbons (GNRs) to form PANI-GNRs composite [42]. As a sulfur host in Li-S batteries, GNRs and PANI improved the mechanical property and electronic conductivity of composite respectively. The synergic effect produced among GNRs, PANI and sulfur contributed to a stable cycling performance and a good rate performance. Liu et al. [43] prepared a nanosulfur@PANI/graphene (nanoS@PANI/G) composite with a sandwich-structure by a one-step method. During the in-situ polymerization of aniline on graphene sheets, sulfur particles were chemically deposited on a PANI layer. The PANI/G network exhibited a high conductivity and flexibility, which ensured a superior cycling performance and rate capability of Li-S batteries, with an initial capacity that even reached 1625 mAh g^−1^ at 0.1 C.

In order to compare the contribution of different conducting polymers to electrochemical performance of Li-S batteries, Wang et al. [45] fabricated different conducting polymers/GO@S composites. When assembled in a Li-S cell, the poly(3,4-ethylenedioxythiophene) (PEDOT)/GO@S composite containing 66.2 wt% sulfur delivered a capacity of 800.2 mAh g^−1^ after 200 cycles (0.5 C), much higher than that of PANI/GO@S. Moreover, the PEDOT/GO@S composite also exhibited a good rate capability, and the capacity remained at 632.4 mAh g^−1^ even at 4 C. The result showed that PEDOT was a better option than PANI to inhibit the shuttling of sulfur in Li-S batteries.

### 2.3. Covalent Bond Compounds

Besides as coating layer and conductive host, PANI can be pyrolyzed to form novel vulcanized polymer cathodes, and the vulcanized-polymeric cathode provides a new strategy for fabricating super stable high-rate Li-S batteries. Tsao et al. [46] polymerized a PANI coating on the surface of sulfur particles, then thermally treated them to produce covalent bonds between S and the aromatic rings of the PANI backbone. The as-prepared novel cathode was labeled as S@h-P, and showed the feature of no free polysulfides, and only lower-order polysulfides on the polymer backbone during redox reaction. Therefore, the S@h-P cathode delivered a high capacity retention of 88% after 200 cycles.

In another work, Yan et al. [44] prepared a nanoporous covalent bond S-containing compound by replacing the H atoms in benzene rings by Cl atoms, and then introducing polysulfide groups, as shown in Figure 2c. In this special sulfur-polyaniline (SPANI) cathode, polysulfide groups were connected between two molecular chains of PANI, which achieved the physical and chemical confinement of sulfur species perfectly. As a result, the SPANI cathode delivered a capacity of 500 mAh g^−1^ after 200 cycles at 1 C.

### 2.4. Separator Modifier/Interlayer

**Figure 3:**
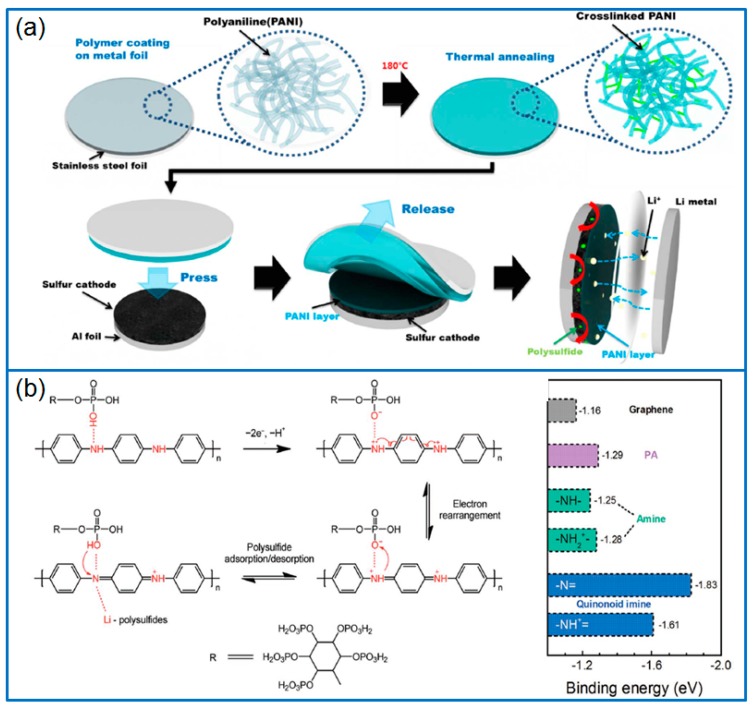
(**a**) Schematic illustration of PANI layer coated on sulfur cathode [48]; (**b**) The formation of quinonoid imines and the adsorption and desorption to polysulfides, and calculated binding energies of Li_2_S_8_ on different matrices or functional groups [49].

PANI can be adopted as a separator modifier or functional interlayer. Chang et al. [47] coated a PANI nanofiber/multiwall carbon nanotube (PANINF/MWCNT) mixture onto a commercial PP separator to prepare functionalized separators. During the reversible charge/discharge process of Li-S batteries, the PANINF/MWCNT coating provided an electron transfer channel, and efficiently inhibited the migration of soluble polysulfides. The Li-S cell containing this functionalized separator retained a high reversible capacity of 709 mAh g^−1^ after 100 cycles at 0.2 C. In addition to chemical polymerization to prepare PANI coatings, a physical method was reported by spin-coating PANI/n-methyl-2-pyrrolidone (NMP) solution onto stainless steel foil [48], as shown in Figure 3a. The as-prepared PANI film was heated to form a crosslinked PANI film, then it was transferred and printed on the top of the GO-S electrode. The PANI interlayer on the top of sulfur cathode improved the electronic conductivity of electrode, and also suppressed the dissolution and diffusion of soluble polysulfides, which enabled a Li-S cell with a high capacity retention of 96.4% for 200 cycles.

### 2.5. Redox Mediator

Besides these traditional functions as coating layer, conductive host and separator modifier/interlayer, the N-containing groups in PANI can also act as highly efficient redox mediators in Li-S batteries. As seen in Figure 3b, during the redox reaction of the sulfur cathode, the quinonoid imine (-NH^+^=/-N=) group was confirmed as a strong adsorption site to anchor LiPSs and promote the redox kinetics of sulfur species [49].

In summary, as a low cost conducting polymer, PANI can be synthesized by chemical oxidative method, interfacial polymerization, electrochemical method, and so on. The structure design is a promising strategy for improving the electrochemical performance of PANI. In Li-S batteries, PANI can be used as coating layer, conductive host, covalent bond compounds, separator modifier/interlayer and redox mediator. From the performance statistics in Table 1, PANI coating greatly improves the electrochemical performance of sulfur/carbon-sulfur cathode. Among the sulfur hosts, hierarchical porous carbon/carbon spheres wrapped with PANI coating exhibited much better performance than the unmodified hosts. For instance, the PANI-coated colloid carbon spheres cathode containing 73 wt.% S achieved the long-term cycling for 2500 cycles at 5 C [26], which broke through the cycling performance of Li-S batteries. When acted as conductive hosts, the hollow PANI sphere@S cathode presented a high reversible capacity of 602 mAh g^−1^ after 1000 cycles at 0.5 C [38], which represents the best performance among various PANI hosts. In addition, PANI can be used to prepared sulfur-containing compounds by forming covalent bonds, and serves as functional separator/interlayer to enhance the cycling stability by retarding the shuttling of polysulfides. Besides these conventional applications of PANI, the quinonoid imine group (-NH^+^=/-N=) in the copolymer of aniline and phytic acid was confirmed as a strong adsorption site to promote the redox reaction of sulfur species [49], which disclosed a new research topic of PANI in Li-S batteries.

## 3. Application of PPy in Li-S Batteries

Like PANI, PPy can be synthesized by using chemical oxidative methods [50,51,52], electrochemical polymerization [53,54,55], template methods [56] and emulsion polymerization [57]. Besides the normal application as a coating layer, conductive host and separator modifier/interlayer, PPy can act as a binder and current collector in Li-S batteries.

### 3.1. Coating Layer

**Figure 4:**
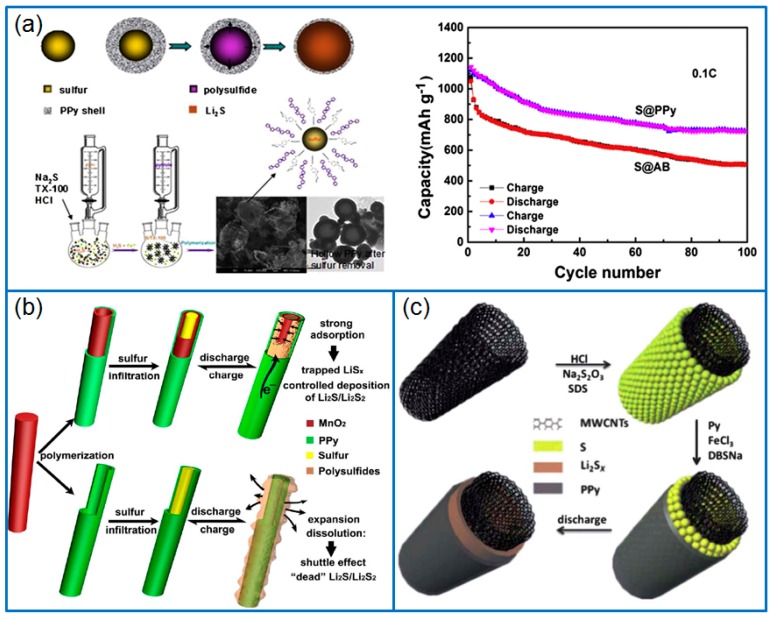
(**a**) Schematic illustration of synthesizing S@PPy composite with core-shell structure and the corresponding cycling stability of Li-S cell [60]; (**b**) Preparation of S/PPy-MnO_2_ ternary composite and the sulfur confining theory [62]; (**c**) Dual core-shell structured MWCNTs@S@PPy composite [66].

As a kind of conducting polymer, PPy can be directly polymerized on the surface of sulfur or sulfur/carbon composites to form coating structures. The PPy layer not only enhances the conductivity of electrodes, but also effectively traps the dissolved polysulfides. Therefore, PPy conductive layers are widely used to improve the cycling stability and rate capability of Li-S batteries. Zhang et al. [58] synthesized a PPy-coated sulfur (PPy@S) composite cathode by polymerizing pyrrole monomers on the surface of nanosulfur particles. The PPy shell layer provided an effective electron conduction path and a strong physical and chemical interaction to confine sulfur. When assembled in a Li-S cell, the initial discharge capacity was 1200 mAh g^−1^ at 0.2 C, and remained at 913 mAh g^−1^ after 50 cycles. Similarly, Yuan et al. [59] also prepared a core-shell structured sulfur/PPy (S/PPy) composite by chemical oxidative polymerization in the presence of surfactant. The thin PPy layer (20~30 nm) provided an electron transfer path and also effectively suppressed the loss of sulfur. Therefore, the S/PPy cathode delivered an enhanced reversible capacity and cycling stability compared to that of a pure sulfur cathode. In addition, Xie et al. [60] synthesized a PO_4_^3−^ doped PPy layer on the surface of as-produced nanosulfur particles, as shown in Figure 4a. Different from the traditional Cl^−^ doping by using FeCl_3_, the PO_4_^3−^ doped PPy enhanced the conductivity of composite. Even at a high sulfur content of 80 wt.%, the Li-S cell exhibited a high initial capacity of 1142 mAh g^−1^ at 0.1 C, and the capacity remained at about 65% after 100 cycles.

Compared to the wrapping by a single PPy layer, the double-layer coating on sulfur will achieve the synergetic effect of different shell layers. For example, Zhou et al. [61] firstly wrapped a PPy layer on as-synthesized sulfur nanoparticles, then covered graphene nanosheets (GS) on S@PPy to form a conductive 3D graphene network. The special S@PPy/GS cathode delivered an initial capacity of 1040 mAh g^−1^ at 0.1 C, and the capacity remained at 537.8 mAh g^−1^ at 0.2 C after 200 cycles. The excellent cell performance was attributed to the buffer effect and sulfur immobilization of the flexible PPy shell. Meanwhile, the outside graphene sheets contributed to the conductivity, and further captured the soluble polysulfides. In addition, Zhang et al. [62] synthesized PPy-MnO_2_ coaxial nanotubes as sulfur host by using MnO_2_ nanowires as oxidant and template, as presented in Figure 4b. Then sulfur was infused into the nanotubes to fabricate a S/PPy-MnO_2_ composite. Through adjusting the content of MnO_2_, the optimized S/PPy-MnO_2_ cathode containing 5 wt.% MnO_2_ presented an ultra-low capacity decay of 0.07% per cycle for 500 cycles at 1 C. The high performance resulted from the synergistic effect of tubular MnO_2_ and PPy, in which, the internal tubular MnO_2_ effectively trapped polysulfides, and the conductive PPy outer layer contributed to the rate capability and cycling performance of Li-S batteries.

Besides the core of sulfur particles, a PPy split-half-tube was synthesized and deposited with sulfur nanoparticles, then a PPy outer layer was wrapped to form a three-layer structured PPy@S@PPy composite [63]. When compared to the S@PPy cathode, the PPy@S@PPy cathode exhibited a higher reversible capacity and capacity retention in Li-S batteries, which further confirmed the sulfur confinement effect of an external PPy layer. Similarly, a tubular PPy was carbonized as a tubular amorphous carbon (TAC) matrix to deposit with nano-sulfur, then, granular PPy was synthesized on the surface of TAC@S as a coating layer [64]. The novel core-shell TAC@S@PPy composite cathode was prepared for Li-S batteries. The external PPy layer ensured the cathode displayed enhanced conductivity and an excellent adsorptivity to dissolved polysulfides.

Among the sulfur hosts of Li-S batteries, carbon nanotubes (CNT) are widely adopted to host sulfur for their excellent electronic conductivity. However, after the cladding of electroactive PPy, the PPy-wrapped CNT/S composite presents an enhanced rate capability and cycling stability. In this field, Wang et al. [65] loaded sulfur into a CNT network firstly, then coated a conductive PPy layer on the surface of S-CNT composite to prepare a S-CNT-PPy ternary composite. Compared to the cathode of S-CNT or S-PPy, the S-CNT-PPy cathode presented much higher reversible capacity.

In another work, Wang et al. [66] deposited sulfur particles on the surface of the carbon nanotubes (MWCNTs), and then wrapped a PPy layer to produce a MWCNTs@S@PPy composite with a dual core-shell structure (Figure 4c).

In this special nanostructure, the elastic PPy layer accommodated the volume change of sulfur, alleviated the dissolution and diffusion of polysulfides. The composite cathode delivered a high initial capacity of 1517 mAh g^−1^ at 200 mA g^−1^, with a stable cycling performance and rate capability. Compared to the wrapping by traditional Cl^–^ doped PPy, Wu et al. [67] synthesized polyethylene glycol (PEG)-doped PPy by adopting PEG400 dopant, and wrapped the PPy/PEG layer on the outer surface of an as-prepared aligned CNT (A-CNT)/S composite. The PEG additive improved the mechanical properties of PPy by forming a stable PPy structure. Moreover, it also adsorbed electrolyte ions and trapped dissolved polysulfides. Therefore, the PPy/PEG-S/A-CNT cathode delivered a high initial capacity of 1355 mAh g^−1^, and remained at 924 mAh g^−1^ after 100 cycles. Even at a high rate of 8 A g^−1^, the reversible capacity still remained at 480 mAh g^−1^ after 100 cycles.

Among the carbon/sulfur composites, the graphene sheets/S and 3D porous carbon/S composite can also be wrapped by PPy layers. Dong et al. [68] prepared graphene-backboned mesoporous carbon (GC) nanosheets to host sulfur (denoted as GCS), then polymerized polypyrrole monomers on the GCS nanosheets to form a GCS@PPy composite. The PPy-coated GCS nanosheet efficiently suppressed the loss of active sulfur. The Li-S cell presented a high discharge capacity and an ultra-long cycling stability. The capacity decay rate was low as 0.05% per cycle after 400 cycles at 1~3 C rate. Different from mesoporous carbon CMK-3, CMK-8 is a kind of 3D cubic mesoporous carbon with conductive framework. It has been widely adopted as an electrode for supercapacitors [69]. Recently, Ma et al. [70] adopted PPy as a wrapping layer to coat CMK-8/sulfur (CMK-8/S) and investigated the performance of the resulting PPY@CMK-8/S cathode. Due to the synergistic effect between the 3D porous carbon matrix and the conductive PPy layer, the reversible capacity of assembled Li-S cell remained at 860 mAh g^−1^ after 100 cycles at 0.2C.

### 3.2. Conductive Host

Besides as coating layer, PPy nanwires can be synthesized by using alkyltrimethylammonium (CTAB) as soft template and serve as a conductive matrix to host sulfur [71]. In the as-prepared S-PPy composite, PPy nanowires served as conducting agent, absorbing agent and distribution agent, which effectively improved the cell performance. Besides the PPy homopolymer, Qiu et al. [72] synthesized conductive poly(pyrrole-co-aniline) (PPyA) copolymer nanofibers as sulfur hosts by using cetyltrimethylammonium chloride (CTAC) as surfactant. Sulfur was then infiltrated into PPyA nanofibers by a heating treatment at 160 °C. The novel PPyA framework served as conducting paths for electron transport, and also provided electrochemical reaction sites to the reversible redox reaction in a Li-S battery. Regarding the design of PPy microstructures, Ma et al. [73] synthesized thin wall hollow spherical structured polypyrrole (T-HSSP) by using a SiO_2_ nanosphere template. The special T-HSSP containing single-layer PPy nanoparticles was used as sulfur host to improve the electrochemical performance of Li-S batteries. It was confirmed that the designed special microstructure facilitated the conduction of ions and electrons, buffered the volume change of sulfur and inhibited the dissolution and migration of polysulfides. In addition, PPy was synthesized on the multi-wall carbon nanotubes (MWCNTs) to form a core/shell structured PPy-MWCNT composite [74]. As a sulfur host, the MWCNTs served as a conductive network. The PPy shell improved the dispersion of sulfur, and provided a strong absorption for polysulfides. The optimized PPy-MWCNT sulfur host containing 25 wt.% PPy delivered a reversible capacity of 725.8 mAh g^−1^ after 100 cycles.

**Figure 5:**
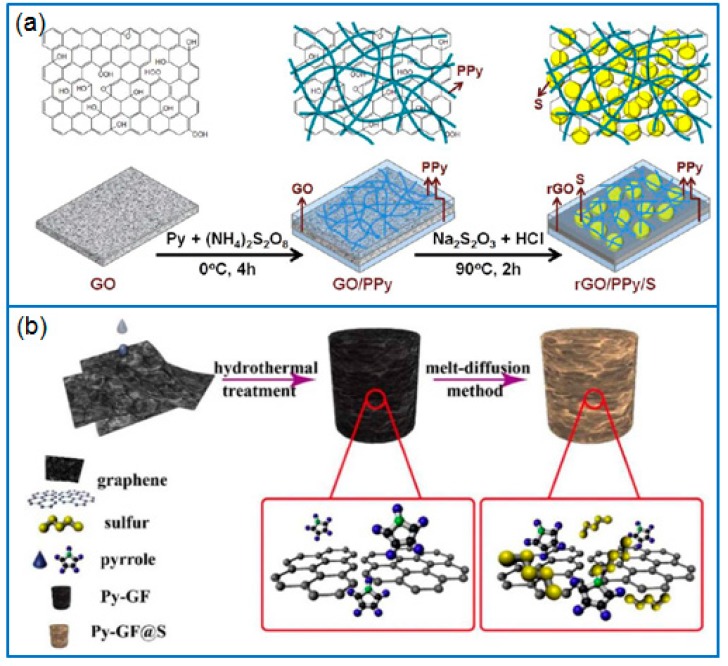
Schematic of preparing (**a**) rGO/PPy/S nanocomposite [75] and (**b**) pyrrole modified graphene aerogel foam (Py-GF) for Li-S batteries [77].

As a popular sulfur host, graphene is often used as a typical 2D matrix to hybridize with PPy. Due to the π-π interaction between pyrrole and graphene, as-produced PPy particles are uniformly dispersed on graphene nanosheets, which further enhances the conductivity of electrodes and the sulfur immobilization. For instance, Qian et al. [75] firstly polymerized PPy on the surface of reduced graphene oxide (rGO), then deposited sulfur to fabricate a ternary hierarchical rGO/PPy/S nanocomposite (Figure 5a). The 3D network structure of rGO/PPy/S improved the conductivity of the cathode and suppressed the loss of active sulfur species. When the sulfur content was 69.43 wt.%, the corresponding Li-S battery presented a stable cycling performance and good rate capability. The capacity remained at 626.7 and 442.1 mAh g^−1^ after 400 cycles at 1 C and 5 C, respectively. Similarly, Zhang et al. [76] also prepared a ternary nano-sulfur/PPy/graphene nanosheet (nano-S/PPy/GNS) composite. The result further confirmed that PPy stuck on a graphene surface effectively suppressed the diffusion of polysulfides. Zhang et al. [77] prepared a 3D pyrrole- decorated graphene aerogel foam (Py-GF) by hydrothermally treating a mixture solution of pyrrole and GO, as shown in Figure 5b. In this modified graphene foam, pyrrole groups connected to graphene sheets by π-π interaction or H-bonding, which trapped dissolved polysulfides and improved the conductivity of the framework, while the 3D graphene framework provided a conductive pathway and accommodated abundant sulfur. When the sulfur loading was 6.2 mg cm^−2^, the capacity of Li-S battery remained at 797.9 mAh g^−1^ after 100 cycles at 0.5 C, and the capacity retention was 81%.

In the preparation of carbon/S cathodes, ball-milling methods are commonly used for their advantages of being simple, eco-friendly and low-cost. This method can also be adopted to prepare PPy/S composites. Xin et al. [78] synthesized a nanotubular PPy by a self-degrading template method, and then prepared a sulfur-PPy (S-PPy) composite by a ball-milling method. Due to the interstitial structure of S-PPy composite, and the adsorption, conductive feature of PPy, the reversible capacity of assembled Li-S cell were maintained at 675 mAh g^−1^ after 150 cycles at 200 mA g^−1^. Based on the simple ball-milling method, Yin et al. [79] also prepared a S/PPy composite with sulfur particles dispersed on a PPy nanowire network. The interconnected conductive PPy network provided an ion transfer path and efficiently captured the dissolved polysulfides, which enhanced the rate capability and cycling stability of Li-S batteries.

### 3.3. Separator Modifier/Functional Interlayer

In the field of modified separators or functional interlayers, electrochemically active PPy has an ionically and electronically conductive feature, which decreases the polarization of sulfur cathodes and enhances the rate capability of Li-S batteries. Moreover, a PPy separator/interlayer effectively inhibits the shuttle effect through a strong adsorption to polar polysulfides.

**Figure 6:**
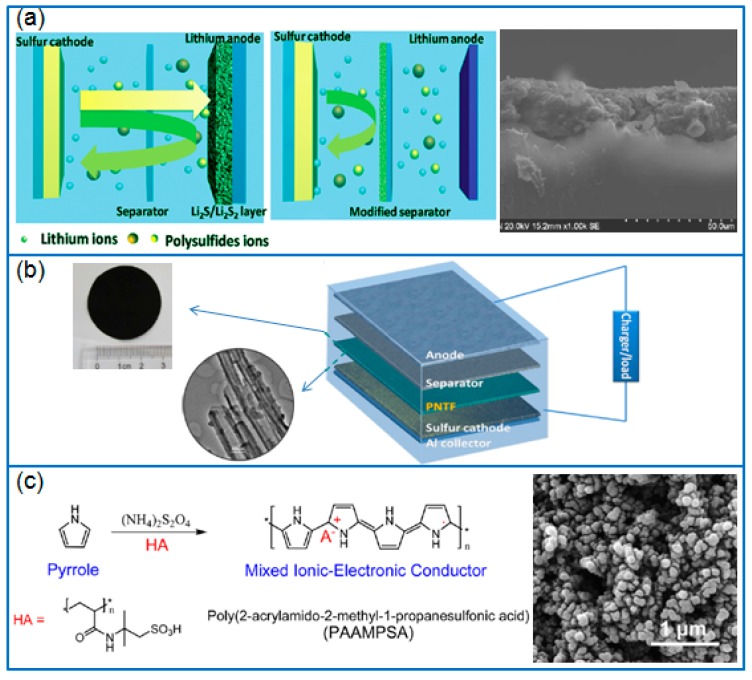
(**a**) The difference of common separator and the modified separator, and corresponding PPy nanotubes modified separator [80]; (**b**) The configuration of Li-S battery with PNTF and corresponding images of PPy nanotube film [81]; (**c**) The synthesis of PAAMPSA-doped PPy mixed MIEC and the corresponding SEM image [82].

In order to compare the function of separator modifiers, Ma et al. [80] prepared PPy nanotubes, PPy nanowires, and a rGO-modified separator respectively by a vacuum filtration method. Using the same cathode of Ketjen black/S, a PPy-modified separator exhibited a lower polarization and a stronger adsorption to polysulfides than a rGO-modified separator (Figure 6a). However, compared to PPy nanowires, the PPy nanotubes-modified separator in Li-S cell presented a higher reversible capacity and cycling stability, and the capacity remained at 801.6 mAh g^−1^ at 0.5 C after 300 cycles. Therefore, nanotubular PPy can be adopted as a suitable separator modifier. Ma et al. [81] prepared a PPy nanotubes film (PNTF) by vacuum filtrating an as-synthesized tubular PPy (T-PPy) solution, as given in Figure 6b. When located between separator and cathode, the PNTF interlayer reduced the polarization of cathode, effectively preventing the dissolution and migration of polysulfides. When the interlayer was combined with a Ketjen black/S cathode (2.5~3 mg cm^−2^ S), the discharge capacity of battery remained at 712 mAh g^−1^ after 300 cycles at 0.5 C.

Besides traditional free-standing functional interlayers, Ma et al. [83] firstly coated a mesoporous carbon CMK-8/S composite slurry on aluminum foil to prepare a cathode, and then fabricated a PPy functional interlayer (PFIL) on the top of this cathode by in situ chemical oxidative polymerization. The PFIL tightly formed on the surface of sulfur cathode prevented the structural damage of the cathode during a long-term charge/discharge, and efficiently suppressed the shuttling of polysulfides. Therefore, the reversible capacity of Li-S cell remained at 703 mAh g^−1^ and 533 mAh g^−1^ after 300 cycles at 1 C and 2 C, respectively.

### 3.4. Binder

In the field of electroactive binders, PPy nanoparticles can be used to form electrically percolating networks by dispersing them in an elastic polyurethane (PU) matrix [84]. The PPyPU binder was used to fabricate flexible sulfur cathodes by dropping a S/C-PPyPU slurry onto a conductive carbon felt. Compared to conventional inactive binders, the novel PPyPU binder changed the fabrication of sulfur cathodes, avoiding hazardous reagents and expensive alumina templates. In order to prepare doped PPy, poly(2-acrylamido-2-methyl-1-propanesulfonic acid) (PAAMPSA) was adopted as a dopant, PAAMPSA-doped PPy (Figure 6c) was synthesized as a kind of mixed ionic-electronic conductor (MIEC) [82]. As a conductor of ions and electrons, sulfur particles were deposited on this MIEC to form a sulfur-MIEC composite, and the S-MIEC cathode exhibited an excellent rate capability and cycling stability.

### 3.5. Current Collector

Besides the conductive binder, PPy films are also used as current collectors. Recently, Li et al. [85] coated a S@PPy composite slurry onto a flexible PPy film prepared by an electrodeposition method, and prepared a free-standing S-PPy cathode. In addition, a PPy nanofiber-coated separator was used in this flexible Li-S battery. The PPy film current collector had a strong adhesion to active the S@PPy cathode, which buffered the volume change of sulfur and effectively anchored the polysulfides. As a result, the resulting flexible Li-S battery delivered a high discharge capacity and cycling stability.

In this section, the syntheses of PPy are introduced briefly. Like PANI, PPy can also be synthesized by chemical oxidative methods, electrochemical deposition and template methods. In the application field, unlike PANI, PPy can act as binder and current collector, which further reflects the high conductivity of PPy. Aside from the superior conductivity, the active pyrrole N groups in PPy also serve as chemical anchoring sites to achieve excellent sulfur confinement. From the cell performance of PPy-based composites in Table 2, the performance of Li-S batteries is related to the thickness of the PPy layer, the sulfur species, sulfur hosts and sulfur loading. 

Compared to conventional PPy doped by Cl^−^, PO_4_^3−^-doped PPy coating layers present a higher reversible capacity and stable cycling performance [60]. In addition, the double-layer coating on sulfur particles exhibits a superior sulfur confinement than a single PPy layer. For example, PPy/graphene sheets hybrid coating layer on sulfur particles [61] achieved a stable cycling for 200 cycles. Furthermore, the PPy-MnO_2_ hybrid coating almost delivered the best performance in the reported works. The initial capacity of the assembled Li-S cell was as high as 1420 mAh g^−1^, and remained at 985 mAh g^−1^ for 200 cycles at 0.2 C [62]. This result shows that PPy and metal oxide layers have an excellent synergistic effect to trap polysulfides. As a conductive host, the hollow spherical structured polypyrrole (T-HSSP) presented the best performance among the reported PPy hosts [73], which reflected the advantages of hollow spherical PPy structures. Therefore, the structure design and hybrid coatings of PPy are the main strategies to improve the electrochemical performance of Li-S batteries.

## 4. Application of PTh and Its Derivatives

Like PANI and PPy, polythiophene (PTh) and its derivatives can be synthesized by using chemical oxidative polymerization [86] and electrochemical methods [87]. Compared to PANI and PPy, PTh and its derivatives have a higher cost [88]. Among these conducting polymers, PEDOT: polystyrene sulfonate (PSS) is a commercial conducting polymer solution, which has been widely reported as coating layer in Li-S batteries for its high conductivity and facile fabrication. In addition, PTh and its derivatives can serve as functional interlayers and binders, or be used for synthesizing sulfur-containing copolymers.

### 4.1. Coating Layer

Similar to the coating layers of PANI and PPy, the conductive PEDOT or PEDOT:PSS coating layer has an excellent electronic and ionic conductivity. When coated on the surface of a sulfur cathode, it will enhance the conductivity of the electrode, buffer the volume change of sulfur and effectively suppress the shuttling of polysulfides.

**Figure 7:**
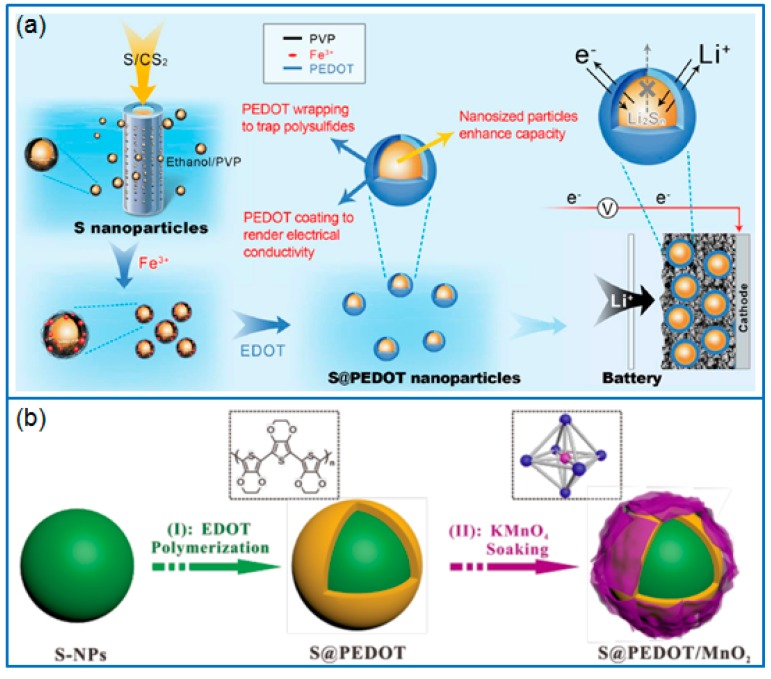
Schematic illustration for preparing (**a**) S/PEDOT nanoparticles and their functions [89]; (**b**) double-layer S@PEDOT/MnO_2_ composite [91].

Chen et al. [89] synthesized nano-S particles (10~20 nm) via a precipitation method and wrapped PEDOT on these S particles to prepare a core/shell structured nano-S/PEDOT composite (Figure 7a) for Li-S batteries. The PEDOT shell layer efficiently prevented the dissolution and diffusion of polysulfides, which delivered an enhanced capacity and cycling stability compared to that of a nano-S cathode. Due to the water solubility, the PEDOT:PSS can be directly used to coat commercial sulfur powder by a wet mixing process [90]. The PEDOT:PSS layer effectively confined the diffusion of lithium polysulfides, and prominently improved the reversible capacity and cycling performance.

Among the carbon-based hosts for sulfur cathodes, Yang et al. [92] prepared a CMK-3 mesoporous carbon/sulfur composite firstly, and then coated a PEDOT:PSS layer on the composite to improve the electrochemical performance of Li-S batteries. Under the protection of the PEDOT:PSS coating layer, the capacity retention of the Li-S batteries increased to ∼80% from ∼70% per 100 cycles, and the Coulombic efficiency was enhanced to 97% from 93%. In another work, GO nanosheets were used as hosts to deposit nanosulfur, then, the PEDOT:PSS solution was added and the GO was reduced. After a vacuum filtration of the mixture suspension, a free-standing sandwiched PEDOT:PSS functionalized S-rGO film was prepared as a binder-free sulfur cathode [93]. The compact PEDOT:PSS-rGO conductive network provided charge transfer paths and buffered the volume change of sulfur. Moreover, the oxygen-containing groups in graphene and the functional groups in PEDOT:PSS layer both suppressed the shuttling of LiPSs by chemical interaction. The novel film cathode delivered a high volumetric capacity of 1432 Ah L^−1^ at 0.1 C, and the capacity retention was as high as 80% for 500 cycles at 1 C.

Due to a large surface area and pore volume, a MIL-101(Cr) metal-organic framework (MOF) was adopted as sulfur hosts, and biomolecule-doped PEDOT:PSS was coated on the MIL-101(Cr)/S composite to prepare a core-shell sulfur cathode [94]. The PEDOT:PSS coating enhanced the conductivity of the cathode, and provided a strong binding to Li_2_S/Li_2_S_2_, which inhibited the diffusion of polysulfides. The special sulfur cathode ensured an excellent cycling stability of Li-S cell, and the capacity remained at 606.62 mAh g^−1^ after 192 cycles at 0.1 C. Besides MOF (MIL-101) host, a kind of Prussian blue analogue (PBA), and sodium iron cyanide (Na_2_Fe[Fe(CN)_6_)]) nanocrystals were adopted as sulfur hosts [95]. As a coating layer, PEDOT was coated on the S@Na_2_Fe[Fe(CN)_6_) composite as a novel cathode. The PBAs nanocrystals provided abundant Lewis acid sites to adsorb the negatively charged polysulfides. Moreover, the PEDOT layer also trapped the polysulfides. As a result, the PEDOT-wrapped PBAs/sulfur cathode maintained a higher reversible capacity (1101 mAh g^−1^) than that (763 mAh g^−1^) of PBAs/sulfur cathode after 100 cycles at 0.1 C.

Besides the PEDOT coating layer, the PEDOT-co-PEG copolymer solution was also used to encapsulate synthesized sulfur particles [96]. This dual-conducting polymer layer provided transport paths for lithium ions and electrons, and retarded the dissolution of soluble LiPSs into the electrolyte. The result showed that a 1 wt.% PEDOT-co-PEG coating layer on sulfur cathode presented a higher reversible capacity and better cycling stability than the pure sulfur cathode. Similar to PEDOT, poly(3,4-ethylenedioxypyrrole) (PEDOP) is a kind of conducting polymers with a lower oxidation potential [97]. Mukkabula et al. [98] wrapped hydroxyl-functionalized carbon nanotubes/S composite by PEDOP layer to suppress the dissolution and diffusion of polysulfides. The result further confirmed that PEDOP layer on the sulfur cathode facilitated the ion transfer at the interface and retarded the migration of soluble polysulfides to the anode, which greatly improved the Coulombic efficiency, reversible capacity and cycling stability of Li-S battery.

Besides the monolayer capping of PEDOT, a double-layer coating on a sulfur cathode will achieve the synergistic effect of two different coating layers. For example, Yan et al. [91] wrapped a PEDOT layer on as-synthesized sulfur particles by a chemical oxidative method, then modified the PEDOT layer with MnO_2_ nanosheets to prepare a S@PEDOT/MnO_2_ composite, as shown in Figure 7b. The PEDOT layer ensured the high conductivity and inhibited the dissolution of polysulfide. In addition, the MnO_2_ layer trapped the LiPSs through chemical adsorption. Hence the S@PEDOT/MnO_2_ cathode maintained a reversible capacity of 827 mAh g^−1^ after 200 cycles at 0.2 C, much higher than that of S@PEDOT cathode (551 mAh g^−1^).

### 4.2. Sulfur-Containing Copolymer

**Figure 8:**
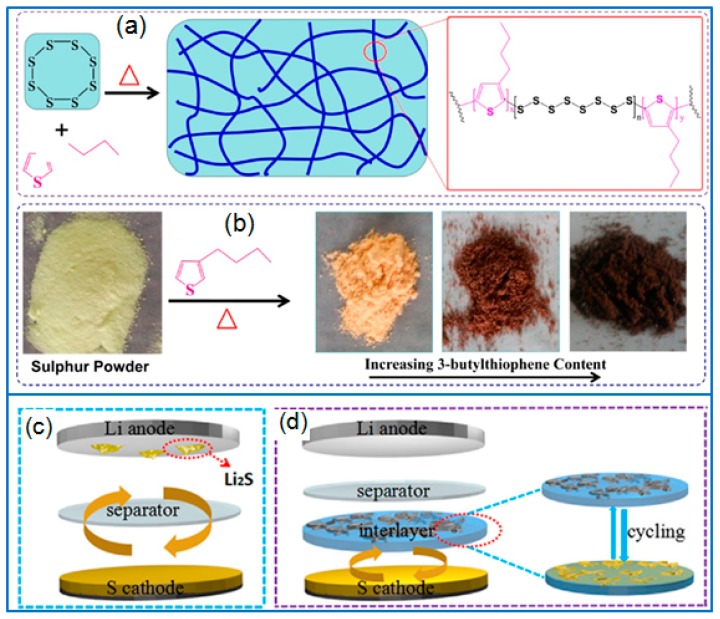
(**a**) The synthesis of S3BT and (**b**) the color change of different samples [100]; The configuration of Li-S cell with (**c**) normal separator and (**d**) the PEDOT:PSS-CNT interlayer [102].

Some polymers containing reactive groups can be used to synthesize sulfur-containing copolymers by radical polymerization [99]. However, the insulation features of sulfur and polymer limit the application of sulfur-containing copolymers. Instead of normal polymers, conducting polymers can enhance the electrical conductivity of sulfur cathode when applied for synthesizing sulfur-containing copolymers. Followed this strategy, Zeng et al. [100] synthesized a sulfur- containing copolymer by using sulfur powders and 3-butylthiophene (3BT) as components, as illustrated in Figure 8a,b. Then a conductive PEDOT: PSS layer was coated on the sulfur-containing copolymer to prepare a capped active sulfur cathode. The enhanced cell performance was mainly attributed to the sulfur confinement derived from the chemical bonds of the S-containing copolymer and the sulfur anchoring role of the PEDOT:PSS layer. In addition, *m*-aminothiophenol was used to synthesize conductive poly(*m*-aminothiophenol) (PMAT). The abundant thiol groups in PMAT enabled the copolymerization between sulfur and PMAT to form a conductive S-PMAT copolymer [101]. Due to the stable covalent bonds formed between thiol groups and sulfur, the chemical confinement of sulfur guaranteed the long-term cycling stability of Li-S batteries. Under the sulfur loading of 1.5 mg cm^−2^, the S-PMAT copolymer cathode exhibited a high reversible capacity and a super-long cycling stability, and the capacity remained at 495 mAh g^−1^ after 1000 cycles at 2 C.

### 4.3. Functional Interlayer

In the configuration design of Li-S batteries, the functional interlayer serves as the second current collector and the adsorption layer of polysulfides to inhibit the shuttle effect [103]. Besides various carbon materials interlayers, conductive PEDOT can be adopted as a functional interlayer to improve the cell performance. Recently, a free-standing PEDOT:PSS-carbon nanotube (CNT) film was prepared by a vacuum filtration method [102]. When placed between the super P/S cathode and separator, the PEDOT:PSS-CNT interlayer effectively suppressed the shuttling of polysulfides by providing a strong chemical absorption (Figure 8c,d). Hence the Li-S cell containing the PEDOT: PSS-CNT interlayer presented a good rate capability and cycling stability, and the capacity remained at 653 mAh g^−1^ after 200 cycles at 0.5 C. Besides preparing free-standing PEDOT:PSS films, commercial PEDOT:PSS aqueous solution can be directly pasted on the super P/S cathode to fabricate a PEDOT:PSS protective layer [104]. Due to the buffer effect and the sulfur confinement of PEDOT:PSS active layer, the coated super-P/S cathode presented an initial capacity of 1061 mAh g^−1^ at 0.2 C. After testing for 100 cycles, the capacity remained at 638 mAh g^−1^, which was much higher than that of a super P/S cathode.

### 4.4. Binder

Conductive PEDOT can serve as a binder in Li-S batteries. For example, Wang et al. [105] prepared a PEDOT-based sulfur electrode with a PEDOT binder and made a comparison with polyvinylidene difluoride (PVDF) binder, and discussed the effect of sulfur sizes and electrolyte solvents. Due to the excellent electrical conductivity and strong adsorption to polysulfides, the PEDOT-containing sulfur cathode presented an enhanced capacity and cycling stability compared to the cathode containing PVDF binder. Moreover, the Li-S cell with PEDOT binder, micrometric sulfur and polyethylene glycol dimethyl ether (PEGDME) electrolyte exhibited the best performance, with a capacity retention of 68% after 100 cycles.

In summary, chemical oxidative polymerization and electrochemical methods can be used to synthesize PTh and its derivatives. Due to the high cost of thiophene/alkyl thiophene monomer, there are few works about PTh and PEDOT in Li-S batteries. However, different from PANI and PPy, commercialized PEDOT:PSS solution facilitates the fabrication of conducting polymer coatings and high-performance sulfur cathodes. About the applications, PTh and its derivatives can act as coating layers, sulfur-containing copolymers, functional interlayers and binders in Li-S batteries. In the existing literature, PEDOT or PEDOT:PSS mostly serve as coating layers for wrapping sulfur cathodes. As given in Table 3, after wrapping with PEDOT, the MOF (MIL-101) and Prussian blue analogues (PBAs) exhibit a superior cycling stability in Li-S batteries. In addition, PEDOT-co-PEG copolymer coating [96] presents a superior sulfur confinement than normal PEDOT. Remarkably, the copolymer of sulfur and thiophene including S3BT copolymer [100] and S-PMAT copolymer [101] achieves an ultra-long cycling performance of even more than 500 cycles. The reversible capacity of a S-PMAT copolymer cathode remains at 495 mAh g^−1^ after 1000 cycles at 2 C, which almost breaks through the performance record of conducting polymers in Li-S batteries. Therefore, the copolymers of sulfur and conductive PTh provide a promising strategy for preparing high-performance Li-S batteries.

## 5. Summary and Perspectives

### 5.1. Summary

With the urgent requirement for high-performance Li-S batteries, lots of conducting polymers have been developed and used as the components for Li-S batteries. In this review, the synthesis of PANI, PPy and PTh are introduced briefly, and their application advances in Li-S batteries are summarized in detail.

**Figure 9:**
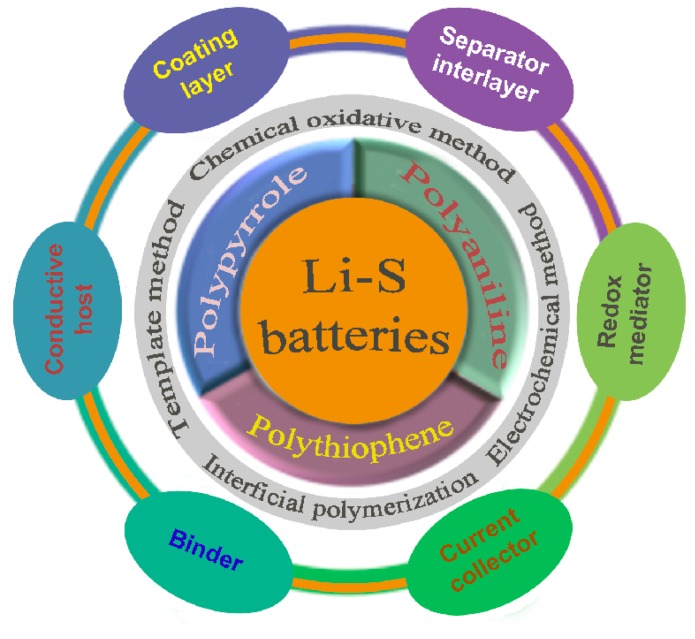
Schematic presentation of the synthesis of conducting polymers and their application in Li-S batteries.

As shown in Figure 9, these conducting polymers can be synthesized by chemical oxidative polymerization, electrochemical methods, interfacial polymerization and template methods. Concerning the applications of conducting polymers in Li-S batteries, we classify their functions into coating layer, conductive host, sulfur-containing compound, functional separator/interlayer, binder, current collector, and so on. In order to make a comparison, the specific application of three polymers in Li-S batteries are summarized in Table 4. Among the reported works on Li-S batteries, most works involve conducting polymer coatings on sulfur particles or carbon/sulfur composites. Compared to conventional conducting polymer coatings, the doped conducting polymer coatings exhibit superior performance. Moreover, the double-layer coating achieves a synergistic effect of different layers, which delivers a better performance than single layers of conducting polymer. In addition to the polymer coating, the sulfur hosts also play an important role in deciding the cell performance. For example, the spherical porous carbon, MOF (MIL-101) and Prussian blue analogues (PBAs) usually embody the essential advantage of sulfur confinement. As conductive hosts, the microstructure design of conducting polymers is a mainstream research direction, for example, synthesizing nanotubes, hollow spheres, and hybridizing them with a carbon matrix. Due to the better conductivity, polythiophene-based sulfur-containing compounds exhibit better cycling stability than the compounds of PANI or PPy. The functional separator/interlayer of conducting polymers can enhance the cycling stability of Li-S batteries. However, the functional separator/interlayer increases the total weight of electrode, which will consume much more electrolytes and reduce the energy density of Li-S batteries.

### 5.2. Perspectives

Despite the great progress achieved in conducting polymers, however, there are still some problems to be solved in the application for Li-S batteries, such as, the microstructure design of conducting polymers, the weak sulfur confinement, the poor performance of sulfur-containing copolymers, and unclear sulfur confinement mechanism. In order to overcome these problems, we propose some strategies to develop conducting polymers for high-performance Li-S batteries:

(1) Developing controllable synthetic techniques for conducting polymers. The species and microstructures of conducting polymers decide their performance and application. Generally speaking, the performance of Li-S batteries depends on the thickness of the coating layer, the types and microstructure of conducting polymers. Therefore, suitable synthetic methods and corresponding doping treatments are always a research topic for conducting polymers.
(2) Designing hybrid wrapping layers based on chemical anchoring mechanisms. Compared to single layers of conducting polymers, double-layer coatings on sulfur cathodes achieve the synergistic effect of different coatings, and greatly improve the cell performance. For example, PPy and MnO_2_ hybrid layers on a sulfur cathode exhibited stable cycling for 200 cycles. Therefore, the combination of conducting polymers with metal oxide layers will become a promising research direction.
(3) Synthesizing high-performance sulfur-containing compounds. Because covalent bonds can be formed between sulfur and conducting polymers, while the covalent bonds also play a crucial role in anchoring sulfur, therefore, various sulfur-containing compounds have been synthesized for Li-S batteries, such as, SPANI, S3BT, and S-PMAT copolymers. Developing sulfur-containing compounds will become a hot topic in the commercialization of conducting polymers and Li-S batteries.
(4) Exploring novel chemical anchoring or electrocatalysis mechanisms of conducting polymers. The performance enhancement of Li-S batteries is mostly attributed to the physical confinement and chemical anchoring. However, the specific interaction mechanism of sulfur confinement is very complicated. For example, the quinonoid imine groups (-NH^+^=/-N=) in the conductive copolymers have an electrocatalytic effect on the redox reaction of sulfur species. Therefore, it is an essential task to explore the relationship between the functional groups of conducting polymers and reaction mechanism of Li-S batteries, which will provide a theoretical guidance to develop novel conductive copolymers.

## Figures and Tables

**Table 1:** The performance of PANI-based composites in Li-S batteries.

Composite	Sulfur Content	Capacity (mAh g^−1^/rate)	Cycling Performance (mAh g^−1^/cycles/rate)	Ref.
S@PANI/GO	54.3 wt.%	1524/0.05 C	875/100/0.2 C 641/300/1 C	[19]
hPANI/S/PANI	N/A	N/A	572.2/214/0.1 C	[20]
PANI@S-C	N/A	1257/0.16 mA cm^−2^	600/100/0.16 mA cm^−2^	[21]
C-PANI–S@PANI	87 wt.% 6 mg cm^−2^	1011/0.2 C	835/100/0.2 C	[22]
CMK3/S-PANI	48 wt.%	1103/1 C	649/100/1 C	[23]
PANI@S-OMC/S	N/A	1626/0.1 C	1338/100/0.1 C	[25]
PANI-assisted S/C nanosphere (PSCs-73)	73 wt.%	N/A	345/2500/5 C	[26]
HPC@S-PANI	N/A	1372/0.2 A g^−1^	494.5/500/2A g^−1^	[27]
MWCNTs-S@PANI	N/A	970.8/0.2 C	545.5/205/0.2 C	[28]
PANI-S/SWNT	85 wt.%	1415/0.2 C	1011/100/0.2 C	[29]
3D CNF/S/PANI	N/A	1074/0.2 C	935/300/0.2 C 552/300/1 C	[30]
GO-S@PANI	75 wt.%	1246/0.5 C	80.43%/500/1 C	[31]
CTAB-GO-S	0.8 mg cm^−2^	970/0.2 C 820/0.5 C 770/1 C	715/300/0.2 C 670/500/0.5 C 570/500/1 C	[32]
NGNS-S-PANI	N/A	1227.3/0.5 C	693/100/0.5 C	[24]
S/PANI	55 wt.%	1095/0.1 C	832/100/0.2 C 609/100/1 C	[33]
S-PANI	65 wt.%	977/1 C	862.7/100/1 C	[34]
S/PANI-coated KB (SPKB)	57 wt.%	1338/0.1 C	675/200/0.1 C	[35]
S/PANI-C(SPC)	2.5 mg cm^−2^	1150/0.2 C	732/100/0.2 C	[36]
C-S@PANI	40 wt.%	1453/0.1 C	948/200/0.1 C 922/200/0.1 C/50 °C 581/200/0.1 C/0 °C	[37]
Hollow PANI sphere@S	62 wt.%	1392.7/0.2 C	602/1000/0.5 C	[38]
hPANIs@ S	N/A	761.8/170 mA g^−1^	601.9/100/170 mA g^−1^	[39]
MWCNT-PANI-G	68 wt.%	1290/0.2 C	784/100/0.2 C	[40]
HCNF@PANI-S	74.4 wt.%	960/0.5 C	535/200/0.5 C	[41]
Sulfur-PANI-GNRs (SPGs)	N/A	673/0.4 C	514/400/0.4 C	[42]
nanoS@PANI/G	N/A	1625/0.1 C	600/100/0.1 C	[43]
PEDOT/GO@S	66.2 wt.%	1195.7/0.5 C	800.2/200/0.5 C	[45]
S@h-P	N/A	341/1 A g^−1^	312/300/1 A g^−1^	[46]
SPANI	65 wt.%	N/A	734/200/0.3 C 600/200/0.6 C 500/200/1 C	[44]
PANINF/MWCNT coated separator	N/A	1020/0.2 C 867/0.5 C 791/1 C	709/100/0.2 C 641/100/0.5 C 612/100/1 C	[47]
PANI-printed on S cathode	N/A	935/1 C	901.3/200/1 C	[48]
NPGO-S	3.3 mg cm^−2^	1114/0.2 C 953/1 C	857.8/100/0.2 C	[49]

**Table 2:** The performance of PPy-based composite in Li-S batteries.

Composite	Sulfur Content	Capacity (mAh g^−1^/rate)	Cycling Performance (mAh g^−1^/cycles/rate)	Ref.
PPy@S	N/A	1200/0.2 C	913/50/0.2 C	[58]
PPy coated S	61.9 wt.%	1039/0.1 C	613/50/0.1 C	[59]
PO4^3−^ doped PPy coated nano-S	N/A	1142/0.1 C	742.3/100/0.1 C	[60]
S@PPy/GS	N/A	1040/0.1 C	537.8/200/0.2 C	[61]
S/PPy-MnO_2_	N/A	1420/0.2 C	985/200/0.2 C	[62]
PPy@S@PPy	65.6 wt.%	801/50 mA g^−1^	554/50/50 mA g^−1^	[63]
Tubular carbon@S@PPy	N/A	1111/335 mA g^−1^	731/100/335 mA g^−1^	[64]
S-CNT-PPy	N/A	1240/50 mA g^−1^	600/40/50 mA g^−1^	[65]
MWCNTs@S@PPy	N/A	1517/200 mA g^−1^	917/60/200 mA g^−1^	[66]
PPY/PEG-S/A-CNT	N/A	1355/0.1 C	924/100/0.1 C	[67]
GCS@PPY	N/A	470/3 C	376/400/3 C	[68]
PPy@CMK-8/S	53.7 wt.%	1099/0.2 C	860/100/0.2 C	[70]
S-PPy physical mixing	40 wt.%	1222	570/20	[71]
S/PPyA	N/A	1285	866/40	[72]
S/T-HSSP	58.4 wt.%	1563.3/0.2 C	892.4/200/0.2 C	[73]
S/PPy-MWCNT(25 wt.% PPy)	49 wt.%	1275/0.1 mA cm^−^^2^	725.8/100/0.1 mA cm^−2^	[74]
rGO/PPy/S	69.43 wt.%	991.5/1 C 537.4/5 C	626.7/400/1 C 442.1/400/5 C	[75]
Nano-S/PPy/GNS	N/A	1415.7/0.1 C	641.5/40/0.1 C	[76]
S/PY-GF	6.2mg cm^−2^	1220/0.2 C 985.8/0.5 C	797.7/100/0.5 C	[77]
S-PPY(ball-milling)	49 wt.%	1178/200 mA g^−1^	675/150/200 mA g^−1^	[78]
S/PPy	N/A	931/0.1 C	502.7/100/0.1 C	[79]
S/Ketjen black	N/A	1110.4/0.5 C	801.6/300/0.5 C	[80]
S/Ketjen black	2.5~3mg cm^−2^	1102/0.5 C	712/300/0.5 C	[81]
CMK-8/S	N/A	719/0.2 C	703/300/1 C 533/300/2 C	[83]
S-MIEC	75 wt.%	968/0.1 C	500/50/1 C	[82]
PPy/S@PPy	1.4mg cm^−2^	1064/0.1 C	848/20/0.1 C	[85]

**Table 3:** The performance of polythiophene-based composites in Li-S batteries.

Composite	Sulfur Content	Capacity (mAh g^−1^/rate)	Cycling Performance (mAh g^−1^/cycles/rate)	Ref.
Nano-S@PEDOT	72 wt.%	1117/400 mA g^−1^	930/50/400 mA g^−1^	[89]
S/PEDOT:PSS	N/A	1100/0.1 C	565.7/50/0.2 C	[90]
PEDOT:PSS-coated CMK3/S	N/A	1140/0.2 C	969/100/0.2 C	[92]
Graphene and PEDOT:PSS coated nano-S (SGP)	N/A	1432 Ah L^−^^1^/0.1 C 1038 Ah L^−1^/1 C	806/500/1 C	[93]
Biomolecule-doped PEDOT:PSS coated MIL-101/S (BPCS)	57.884 wt.%	1567.74/0.1 C	606.62/192/0.1 C	[94]
S@Na_2_Fe[Fe(CN)_6_)]@PEDOT	82 wt.%	1291/0.1 C 683/5 C	1101/100/0.1 C 544/200/5 C	[95]
PEDOT-co-PEG coated sulfur (1 wt.% polymer)	N/A	1619/0.2 C	1002/100/0.2 C	[96]
S/MWCNTols/PEDOP	70 wt.%	1611/0.1 C	624/200/0.1 C	[98]
S@PEDOT/MnO2	87 wt.%	1150/0.2 C	827/200/0.2 C 545/200/0.5 C	[91]
S3BT copolymer	70 wt.%	1362/0.1 C	682/500/1 C	[100]
S-PMAT copolymer	1.5mg cm^−2^	1240/0.1 C 600/5 C	495/1000/2 C	[101]
PEDOT:PSS-CNT interlayer	42 wt.%	921/0.5 C	653/200/0.5 C	[102]
PEODT:PSS-coated S cathode	0.75~0.98 mg cm^−2^	1189/0.1 C	790/50/0.1 C	[104]
PEDOT binder/commercial sulfur/PEGDME	50 wt.%	850	578/100	[105]

**Table 4:** The comparison of three conducting polymers in Li-S batteries.

	PANI	PPy	PTh and PEDOT
Coating layer	Most works	Most works	Most works
Conductive host	Most works	Most works	No works
Separator modifier	Few works	Few works	No works
Functional interlayer	Few works	Few works	Few works
Sulfur-containing copolymer	Few works	No works	Few works
Binder	No work	Few works	Few works
Current collector	No work	Few works	No works
Redox mediator	One work	No work	No work
Advantages	Low cost, facile preparation, widely used	Facile preparation, widely used, high conductivity	Commercialized, easy to fabricate
Shortcomings	Poor conductivity	Expensive	Hard to synthesize
